# An Image-Based Genetic Assay Identifies Genes in T1D Susceptibility Loci Controlling Cellular Antiviral Immunity in Mouse

**DOI:** 10.1371/journal.pone.0108777

**Published:** 2014-09-30

**Authors:** Juan Liao, Humberto B. Jijon, Ira R. Kim, Gautam Goel, Aivi Doan, Harry Sokol, Hermann Bauer, Bernhard G. Herrmann, Kara G. Lassen, Ramnik J. Xavier

**Affiliations:** 1 Broad Institute of Massachusetts Institute of Technology and Harvard University, Cambridge, Massachusetts, United States of America; 2 Center for Computational and Integrative Biology, Massachusetts General Hospital, Harvard Medical School, Boston, Massachusetts, United States of America; 3 Gastrointestinal Unit, Center for the Study of Inflammatory Bowel Disease, Massachusetts General Hospital, Harvard Medical School, Boston, Massachusetts, United States of America; 4 Department of Medicine, Division of Gastroenterology, University of Calgary, Calgary, Alberta, Canada; 5 Gastroenterology Department, Saint Antoine Hospital, Assistance Publique Hôpitaux de Paris, Paris, France; 6 Max Planck Institute for Molecular Genetics, Berlin, Germany; University of Cincinnati College of Medicine, United States of America

## Abstract

The pathogenesis of complex diseases, such as type 1 diabetes (T1D), derives from interactions between host genetics and environmental factors. Previous studies have suggested that viral infection plays a significant role in initiation of T1D in genetically predisposed individuals. T1D susceptibility loci may therefore be enriched in previously uncharacterized genes functioning in antiviral defense pathways. To identify genes involved in antiviral immunity, we performed an image-based high-throughput genetic screen using short hairpin RNAs (shRNAs) against 161 genes within T1D susceptibility loci. RAW 264.7 cells transduced with shRNAs were infected with GFP-expressing herpes simplex virus type 1 (HSV-1) and fluorescent microscopy was performed to assess the viral infectivity by fluorescence reporter activity. Of the 14 candidates identified with high confidence, two candidates were selected for further investigation, *Il27* and *Tagap*. Administration of recombinant IL-27 during viral infection was found to act synergistically with interferon gamma (IFN-γ) to activate expression of type I IFNs and proinflammatory cytokines, and to enhance the activities of interferon regulatory factor 3 (IRF3). Consistent with a role in antiviral immunity, Tagap-deficient macrophages demonstrated increased viral replication, reduced expression of proinflammatory chemokines and cytokines, and decreased production of IFN-β. Taken together, our unbiased loss-of-function genetic screen identifies genes that play a role in host antiviral immunity and delineates roles for IL-27 and Tagap in the production of antiviral cytokines.

## Introduction

Genome-wide association studies (GWAS) have revolutionized the study of human genetics and uncovered numerous disease susceptibility genes and loci over the past decade [1]–[5], although the mechanism of how these disease-associated loci/genes may contribute to the pathogenesis of complex diseases remains largely unknown. Type 1 diabetes (T1D) is a multifactorial disorder caused by interactions between genetic and environmental factors [6]. T1D is an autoimmune disorder characterized by destruction of insulin-producing β cells in the pancreatic islets. Previous studies have suggested a role for viruses in T1D susceptibility [7]. Loss or death of β cells can be achieved by direct targeting of cytotoxic T cells against virally infected β cells, or indirectly by inflammation from unrestrained innate immunity [8], [9]. The latter mechanism has been well illustrated by the T1D-associated gene melanoma differentiation-associated gene 5 (*MDA5*), also known as interferon-induced helicase 1 (*IFIH1*), which acts in antiviral defense [10]. The T1D-associated polymorphism in *IFIH1*, rs1990760 or Thr946Ala, has been demonstrated in multiple data sets following an initial report by Smyth *et al.* [10]. A subsequent study showed that individuals homozygous for this risk allele had significantly higher *IFIH1* basal expression and as a consequence, upon infection, cells were highly activated and produced more inflammatory cytokines and chemokines [11]. A recent study identified a chemically induced mutation in *Ifih1* in mouse, which results in constitutive activation of Mda5 and continuous production of type I interferons accompanied by systemic inflammation [12]. It is currently unclear if additional T1D-associated genes alter susceptibility to virus infection and antiviral defense.

Integrity of host immunity, both innate and adaptive, is central to antiviral defense. Host immunity is first triggered by the immediate innate response, which usually starts with recognition of viral cellular components known as pathogen-associated molecular patterns (PAMPs) by host pathogen recognition receptors (PRRs) [13]–[15]. Macrophages, an innate immune cell type that responds to infections and regulates cellular responses, express various PRRs that are specific to PAMPs associated with different pathogens. Viral PAMPs are recognized by several PRRs including the Toll-like receptors (TLRs), retinoic acid-inducible gene I (RIG-I)-like receptors (RLRs), and a number of cytosolic dsDNA sensors [16]–[20]. In particular, TLR2, a transmembrane protein expressed on the cell surface, senses viral surface glycoproteins [21]. Upon entry, the unmethylated CpG motif, which is a signature of bacterial and viral genomes, is detected by intracellular TLR9 [22]. TLR3 recognizes dsRNA longer than 30 bp, which has been suggested to be an erroneous byproduct during massive viral DNA replication [23]. In addition, another class of cytoplasmic dsRNA sensors, RIG-I and MDA5, recognizes lengths of dsRNA and 5′-triphosphate ssRNA that are absent from most cytosolic mammalian RNA [24], [25]. RIG-I and MDA5 independently signal downstream to an adaptor molecule named mitochondrial antiviral signaling (MAVS). MAVS subsequently activates transcription factors including NF-κB, IRF3, and IRF7, which translocate to the nucleus and upregulate expression of type I interferons (IFN-α and IFN-β) and interferon-stimulated genes (ISGs) [26], [27]. Additional evidence has also suggested participation of recently characterized cytosolic dsDNA sensors, including RNA polymerase III [28], interferon inducible protein 16 (IFI16) [29], and DNA-dependent activator of interferon regulatory factor (DAI) [30] in induction of type I IFNs after viral infection. Type I IFNs and hundreds of ISGs function synergistically to establish an active antiviral state in host cells [27], [31], [32].

Herpes simplex virus type 1 (HSV-1) is a dsDNA virus that belongs to the herpes virus family Herpesviridae. HSV-1 features high infectivity of macrophages and is recognized by multiple innate defense pathways including TLR- and RIG-I/MDA5-dependent pathways [33]. The broad defense pathways triggered by HSV-1 as well as its high infectivity of macrophages make it well suited as a model virus to study antiviral pathways. Here we report an image-based high-throughput genetic screen to identify uncharacterized genes controlling cellular antiviral immunity and characterize roles for IL-27 and Tagap in antiviral defense. Further investigation holds the promise of finding strategies that enhance the antiviral activity of these genes and developing novel effective antiviral drugs that work to combat complex diseases as well.

## Results

### IFN-γ Activates Cellular Antiviral Response in RAW 264.7 Cells

**Figure 1:**
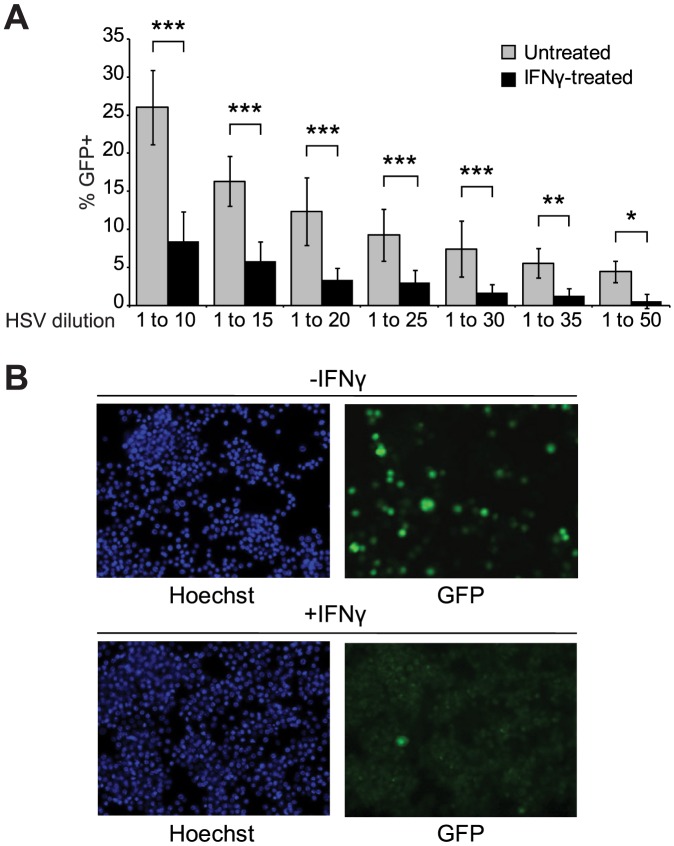
Dose-dependent HSV-GFP infectivity in untreated and IFNγ-treated RAW 264.7 cells. **(A)** RAW 264.7 cells were seeded in 96-well glass-bottom plates at a density of 0.3×10^5^ cells/well and either left untreated or treated with IFN-γ (10 ng/ml) overnight before HSV-GFP infection at the indicated dilution of viral stock. Note that when the viral stock was diluted 1 to 10, the MOI was 0.5. The average percentage of GFP-positive cells was calculated from six individual images per treatment condition. Data shown are representative of 3 independent experiments. ***P<0.001; **P<0.01; *P<0.05 by two-way ANOVA with Bonferroni post-test. **(B)** Representative images of HSV-GFP infection at 1∶25 dilution of the viral stock. Nuclei stained with Hoechst are shown in blue and HSV-GFP infected cells are shown in green.

IFN-γ is a type II class of interferon and critical player in innate and adaptive immunity against viral infection [34], [35]. It has also been shown to play an important role in suppression of HSV-1 infection and reactivation from latency [36], [37]. Therefore, we selected HSV-1 as a model viral pathogen to delineate the role of cellular genes in IFNγ-mediated antiviral immunity. First, we examined whether IFN-γ was capable of activating cellular antiviral activity in RAW 264.7 cells, a mouse leukaemic monocyte macrophage cell line, upon HSV-1 infection. RAW 264.7 cells were pre-treated with IFN-γ for 16 hours before infection with recombinant HSV-1 expressing GFP (hereafter HSV-GFP). The GFP expression cassette was under the control of the Egr-1 promoter and inserted into the intergenic region between the viral *UL3* and *UL4* genes [38]. Previous studies have demonstrated that the presence of the GFP cassette does not have an impact on viral growth or viral infectivity in cell culture or animal models [38]. RAW 264.7 cells were infected with HSV-GFP and the efficiency of viral infection and replication was determined by the percentage of GFP-positive cells 16 hours post infection (Figure S1). Dose-dependent viral infectivity was confirmed by proportional change in the percentage of GFP-expressing cells (Figure 1A). A 3- to 4-fold reduction in HSV-GFP infectivity was observed in IFNγ-treated cells compared to untreated cells (Figure 1A and 1B). These results demonstrate that IFN-γ activates the antiviral machinery in RAW 264.7 cells to suppress HSV-1 replication.

### An Image-based shRNA Screen Identifies Gene Candidates with anti-HSV Activity

To identify T1D-associated genes that are required for antiviral immunity, we developed an image-based high-throughput assay to measure the contribution of individual genes to IFNγ-mediated inhibition of viral infection. First, optimal infectivity of HSV-GFP was titrated as shown in Figure 1A to limit the infection efficiency to 5–10% with IFN-γ priming. Presence of irrelevant non-targeting shRNA against lacZ or luciferase did not affect the IFNγ-mediated inhibition of HSV-GFP infection (Figure S2A). However, in RAW 264.7 cells transduced with positive control shRNAs to knock down gene expression of IFN-γ receptor 1 (*Ifngr1*), 2 (*Ifngr2*) or *Ticam2*, the antiviral control by IFN-γ was greatly compromised and a 3- to 4-fold increase of infection efficiency was observed (Figure S2A and S2B).

**Figure 2:**
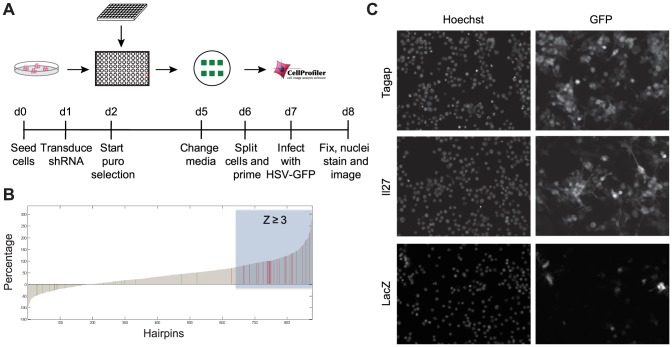
Loss-of-function genetic screen identifies positive regulators of antiviral responses. **(A)** Protocol schematic of the primary genetic screen. **(B)** Ranked range normalization plot of the 827 shRNA of interest and controls. Range normalized HSV-GFP infection efficiency for each individual shRNA-transduced well, expressed as percentage within range 0 to 1, is ranked from low to high and displayed. Green bars indicate normalized irrelevant shRNA controls and red bars indicate normalized positive controls. Grey bars represent normalized shRNA targeting individual genes within T1D susceptibility loci. Location of shRNAs with a Z score equal or greater than 3 is indicated by the area shaded in blue. **(C)** Representative images are shown for two high-scoring shRNAs that target *Tagap* and *Il27* respectively, and one irrelevant shRNA that targets lacZ. Hoechst nucleic acid stain indicates the total cells per microscopic field and GFP indicates the virally infected cells.

Using the conditions described above, a primary genetic screen was performed in RAW 264.7 cells in 96-well plate format with in-plate positive and negative shRNA controls in each plate. A lentivirus-based shRNA library containing 827 lentivirally encoded shRNAs with an average of 5 independent shRNAs targeting each of the 161 genes within T1D susceptibility loci (see Materials and Methods) was obtained from The RNAi Consortium (Table S1). RAW 264.7 cells were transduced with lentiviruses carrying a single shRNA and puromycin was added to select for transductants. The surviving transduced cells were allowed to proliferate for 96 hours before stimulation with IFN-γ before HSV infection (Figure 2A). 16 hours post infection, the HSV infection efficiency for each individual shRNA-transduced well was measured on an automated fluorescent microscope. The percentage of GFP-expressing cells was normalized based on effect size by scaling between 0 (median of wells transduced with irrelevant shRNA) and 1 (median of wells transduced with positive control shRNA) for each individual plate (Figure 2B). After pooling all range-normalized data together, a Z score for each well was calculated from the distribution of wells transduced with irrelevant shRNA. Individual genes were considered to score as positive regulators if 2 or more different shRNAs achieved a Z score equal or greater than 3 standard deviations from the mean for a given gene. Using these criteria, 34 genes were identified from the primary screen (Table S2) after exclusion of shRNAs that caused cytotoxicity or whose targets were not expressed in RAW 264.7 cells.

**Figure 3:**
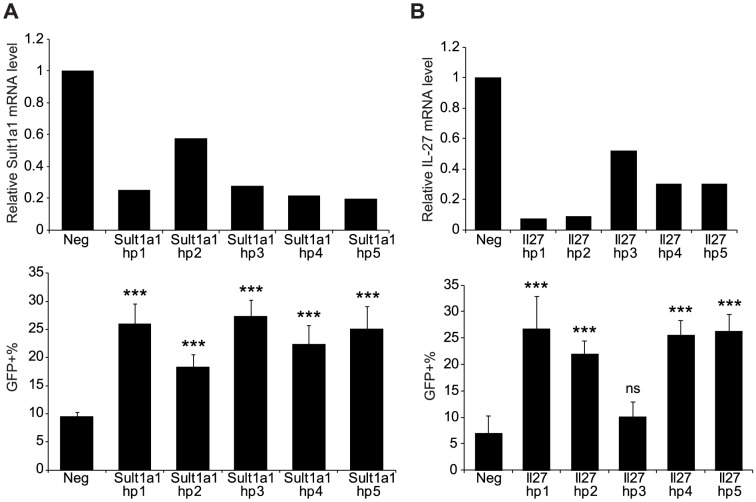
The relative expression level of candidate genes is correlated with observed antiviral phenotype. Upper panels: mRNA expression of *Sult1a1* **(A)** and *Il27* **(B)** in irrelevant shRNA-transduced cells compared to shRNAs against the indicated gene measured by qRT-PCR. *Gapdh* was used as internal control for normalization. The results shown are the average from two independent experiments. Lower panels: percentages of GFP-positive cells after HSV-GFP infection in cells previously transduced with irrelevant shRNA (lacZ and luciferase) or targeting shRNA for *Sult1a1* **(A)** or *Il27* **(B)**. Data shown are representative of 3 independent experiments. ns, not significant; ***P<0.001 compared to negative control by one-way ANOVA with Dunnett's post-test.

The screen was repeated with all shRNAs that scored in the primary screen, and 14 genes were found to have high reproducibility: *Cdk2*, *Ciita*, *Dtx3*, *Esyt1*, *Gca*, *Il27*, *Plxna3*, *Prkd2*, *Rbm17*, *Skap2*, *Tagap*, *Tyk2*, *Sult1a1*, and *Clec2d* (Table 1). Representative images are shown in Figure 2C for shRNA targeting *Tagap* and *Il27*. To further validate the candidate genes, we measured the level of knockdown by the shRNAs against the 14 candidates using quantitative real-time PCR (qRT-PCR). For the majority of the candidates, the targeting shRNA resulted in more than 60% reduction in mRNA levels compared to irrelevant nontargeting shRNA (Figure S3). 10 of the candidate genes displayed a direct correlation between the extent of shRNA knockdown and a corresponding increase in HSV replication, suggesting a gene dosage effect. The results for all 5 shRNAs targeting *Sult1a1* are shown as an example (Figure 3A). The rest of the candidates did not show a graded response to shRNA knockdown, suggesting that for these genes there is a threshold expression level required for the gene to exert its effect (Figure 3B). These results strongly suggest that the increased HSV infectivity is the result of reduced gene expression by targeting shRNAs and that these genes are involved in antiviral immunity of RAW 264.7 cells.

**Table 1:** Candidate genes identified from genetic screen.

Symbol	NCBI Gene ID	Accession number	Preferred Gene Name
*Cdk2*	12566	NM_016756.4	cyclin-dependent kinase 2
*Ciita*	12265	NM_007575.1	class II transactivator
*Dtx3*	80904	NM_030714.2	deltex 3
*Esyt1*	23943	NM_011843.2	extended synaptotagmin-like protein 1
*Gca*	227960	NM_145523.2	grancalcin
*Il27*	246779	NM_145636.1	IL27 p28 subunit; interleukin 30
*Plxna3*	18846	NM_008883.2	plexin 3
*Prkd2*	101540	NM_178900.2	protein kinase D2
*Rbm17*	76938	NM_152824.1	RNA binding motif protein 17
*Skap2*	54353	NM_018773.2	SKAP55 homologue; Srcassociated adaptor protein
*Tagap*	72536	NM_145968.1	T cell activation Rho GTPase activating protein
*Tyk2*	54721	NM_018793.2	tyrosine kinase TYK2
*Sult1a1*	20887	NM_133670.1	Sulfotransferase, 1A, phenol-preferring, member 1
*Clec2d*	93694	NM_053109.1	osteoclast inhibitory lectin

### A Subset of Candidate Genes are Induced by IFN-γ Priming and HSV Infection

**Figure 4:**
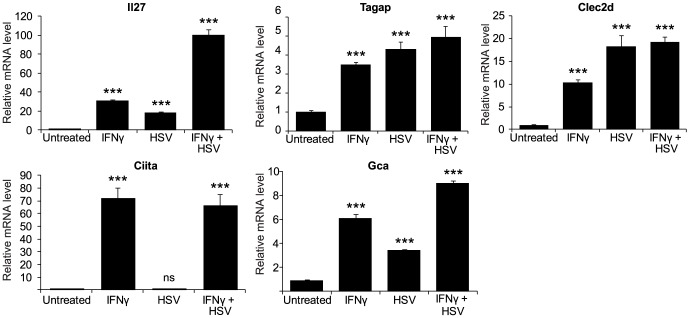
Induction of candidate gene expression by IFN-γ and HSV-GFP infection. Cells were seeded and either left untreated or treated with IFN-γ (10 ng/ml) for 16 h. HSV-GFP (MOI = 0.5) was used to infect the cells as indicated above. qRT-PCR was performed to measure the relative expression of all candidate genes relative to the untreated control. *Gapdh* was used as internal control for normalization. Data shown are results from 3 independent experiments. ***P<0.001 compared to untreated control by one-way ANOVA with Dunnett's post-test.

Since the antiviral state of host cells is initiated by pre-treatment with IFN-γ, we next sought to determine whether the candidates identified from the primary screen were also IFNγ-induced genes. The level of gene expression with or without IFN-γ priming, and upon HSV infection, was measured by qRT-PCR. Among all candidate genes, 9 genes were constitutively expressed and their expression was not altered by either IFN-γ stimulation or HSV infection. 5 candidate genes were transcriptionally induced by IFN-γ stimulation and potentially belong to the group of ISGs (Figure 4). Among the 5 genes, expression of *Il27*, *Tagap*, *Clec2d*, and *Gca* could also be independently induced by HSV infection (Figure 4). This result suggests that these genes are responsive to viral infection and may play a role in viral detection or viral defense in host cells.

### IL-27 and Tagap Positively Regulate Anti-Viral Responses against HSV Infection

**Figure 5:**
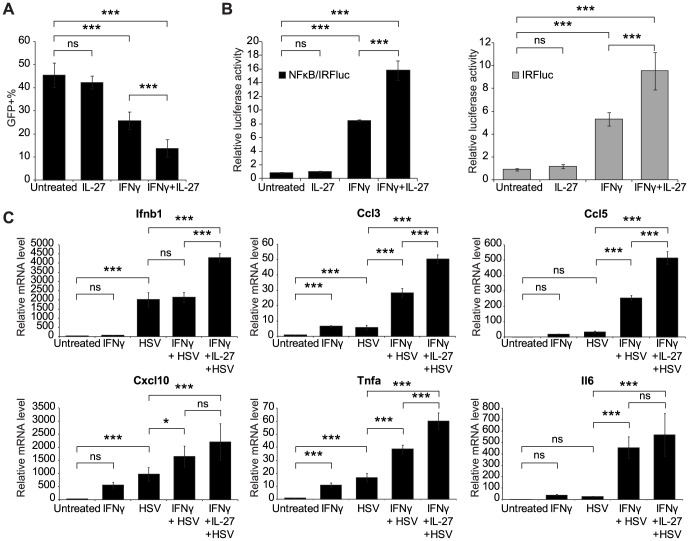
IL-27 functions cooperatively with IFN-γ to suppress the infection of HSV-GFP. **(A)** Cells were seeded in 96-well glass-bottom plates and either left untreated or treated with recombinant IL-27 alone (50 ng/ml), IFN-γ alone (10 ng/ml) or IL-27 + IFN-γ for 16 h. After infecting cells with HSV-GFP overnight, cells were fixed, stained with nuclear acid stain and subjected to imaging. Percentage of GFP-positive cells in each treatment group was calculated and graphed. Data shown are results from 4 independent experiments. **(B)** NF-κB/IRF and IRF reporter cells were seeded and treated as in (A). After stimulating with IL-27 and/or IFN-γ for 16 h, cells were lysed and luciferase activities measured. The reporter luciferase activity in untreated cells is normalized to 1. Data shown are results from 3 independent experiments. **(C)** Cells were seeded and treated as in (A). After infecting cells with HSV-GFP for 6 h, RNA was isolated and qRT-PCR was performed to examine the relative expression levels of downstream effector genes. All expression levels are relative to the level of untreated control and *Gapdh* was used as internal control for normalization. Data shown are results from 3 independent experiments. ns, not significant; ***P<0.001; *P<0.05 by one-way ANOVA with Bonferroni post-test.

Of the 14 validated candidates, we chose to focus further investigation on IL-27 and Tagap due to (1) the strong correlation between phenotype and levels of gene expression, (2) the number of individual shRNAs that led to the desired phenotype, (3) potential biological interest, and (4) availability of animal models. IL-27 is a member of the IL-12 family of cytokines and is mainly secreted by activated antigen-presenting cells (APCs) such as dendritic cells and macrophages [39]. IL-27 has a well-characterized role in T cell differentiation [40] and has been recently reported to have antiviral properties [41]–[43]. To directly assess the antiviral activity of IL-27 against HSV infection, RAW 264.7 cells were treated with recombinant IL-27 (50 ng/ml) either alone or in combination with IFN-γ. 24 hours later, the cells were then infected with HSV-GFP, fixed, and imaged as described above. IL-27 treatment alone did not result in an inhibitory effect on viral infectivity and priming with IFN-γ decreased the viral infectivity by 46% (Figure 5A). Interestingly, the combination of IFN-γ and IL-27 had an additive effect and resulted in the greatest antiviral activity.

Upon viral infection, host cells sense viral components through PRRs and activate antiviral signaling cascades, including the NF-κB pathway and IFN-responsive pathways. IRF3 plays an essential role in this process. Upon activation, IRF3, along with its binding partner IRF7, translocates into the nucleus and binds target DNA through its DNA-binding domain to activate transcription of genes including *IFNα* and *IFNβ* [44], [45]. Production of IFNs further stimulates the activity of other interferon-induced and interferon-stimulated genes in a positive feedback mechanism. Therefore, activity of NF-κB and IRF3/7 is an indicator of antiviral immunity in host cells. To determine whether the inhibitory effect of IL-27 to HSV infection is achieved through NF-κB, IRF3/7, and IFN pathways, luciferase-based IRF and NF-κB/IRF reporter cells were generated in RAW 264.7 cells and the effect of IL-27 treatment was measured by luciferase assay. Treatment of IL-27 alone did not activate either IRF or NF-κB/IRF activity (Figure 5B). However, consistent with the results shown in Figure 5A, co-stimulation of IFN-γ and IL-27 greatly enhanced the activities of both reporters (Figure 5B). Furthermore, to show direct evidence of IFN-γ and IL-27-induced antiviral activity, we examined expression of *Ifnb1* as well as proinflammatory chemokines and cytokines in cells infected with HSV in the presence or absence of IFN-γ and IL-27. Pre-treatment of cells with IFN-γ before HSV infection initiates antiviral signaling cascades that lead to general production of type I interferons, proinflammatory chemokines and cytokines (Figure 5C). *Ifnb1* and *Cxcl10*, an IFN-induced gene, were highly induced in unprimed RAW 264.7 cells upon HSV-1 infection. *Ccl3*, *Ccl5*, *Tnfa*, and *Il6* were expressed at modest levels in unprimed RAW 264.7 cells upon infection, whereas pre-treatment of cells with IFN-γ strongly enhanced HSV1-induced gene expression. Importantly, with the exception of *Cxcl10* and *Il6*, the response of gene expression was augmented by addition of IL-27. These data suggest that administration of IL-27 helps initiate an antiviral signaling cascade and enhances the immunomodulatory activity of IFN-γ in a positive feedback mechanism during viral infection.

To demonstrate the broad antiviral activity of IL-27, we infected IRF and NF-κB/IRF reporter cells with Sendai virus, a ssRNA virus recognized by RIG-I-mediated signaling pathways [46]. IL-27 alone is capable of suppressing Sendai virus infection by enhancing the activities of IRF3 and NF-κB (Figure S4).

**Figure 6:**
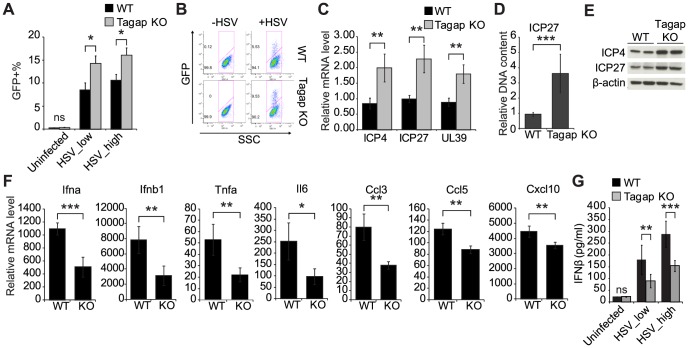
Tagap deficiency results in increased susceptibility to HSV-GFP infection in macrophages. (**A and B**) BMDMs derived from WT or *Tagap^−/−^* mice were infected with HSV-GFP at a MOI of 0.2 (HSV_low) or 0.5 (HSV_high). After 12 h, cells were harvested and stained for GFP. Experiments were performed with a total of 4 mice per genotype. Representative flow plots are shown in (B). ns, not significant; *P = .0463 for HSV_low; *P = 0.0370 for HSV_high. **(C)** BMDMs were infected with HSV-GFP at a MOI of 0.5. After 10 h, relative mRNA levels of three viral genes (*ICP4*, *ICP27*, and *UL39*) were measured in WT versus *Tagap^−/−^* BMDMs. *Gapdh* was used as internal control for normalization. n = 2 with a total of 4 mice per genotype. **P = 0.0032 for ICP4; **P = 0.0013 for ICP27; **P = 0.0014 for UL39. **(D)** BMDMs were infected with HSV-GFP at a MOI of 0.5. After 16 h, DNA was isolated and the relative DNA content of ICP27 in WT versus *Tagap^−/−^* BMDMs was measured. Rplp0 DNA content was used as internal control for normalization. n = 2 with a total of 4 mice per genotype. ***P<0.0001. **(E)** BMDMs were infected with HSV-GFP at a MOI of 0.5. After 24 h, Western blotting was performed using antibodies specific to viral proteins ICP4 and ICP27. β-actin was used as loading control. Representative image from two independent experiments is shown. **(F)** BMDMs were infected as in C. After 3 or 6 h, RNA was isolated to measure the relative expression of pro-inflammatory cytokines or chemokines, respectively. n = 2 with a total of 4 mice per genotype. ***P = 0.0006 for Ifna; **P = 0.0052 for Ifnb1; **P = 0.0063 for Tnfa; *P = 0.0123 for Il6; **P = 0.0014 for Ccl3; **P = 0.0011 for Ccl5; **P = 0.0052 for Cxcl10. **(G)** BMDMs were infected as in (A). After 12 h, culture supernatant was collected and ELISA was performed to measure the amount of secreted IFN-β. n = 3 with a total of 6 mice per genotype. ns, not significant; **P = 0.0094 for HSV_low; ***P = 0.0003 for HSV_high. All statistical comparisons were performed by unpaired two-tailed t-tests.

Unlike IL-27, Tagap has no prior link to IFN signaling or antiviral immunity. GWAS has identified susceptibility loci in *Tagap*, highlighting its role in innate immunity and pathogenesis of autoimmune diseases [47], [48]. To investigate the role of Tagap in antiviral signaling, we generated *Tagap*-deficient macrophages by differentiating bone marrow-derived macrophages (BMDMs) from *Tagap*-deficient mice in M-CSF-supplemented media. BMDMs were then infected with two doses of HSV-GFP. After 24 hours, cells were harvested, stained with APC-conjugated GFP antibody, and analyzed by flow cytometry. *Tagap*-deficient (*Tagap* ^−/−^) BMDMs were more susceptible to HSV infection (Figure 6A and 6B). To further demonstrate increased HSV-GFP infectivity in *Tagap* ^−/−^ BMDMs, intracellular HSV-1 RNA was measured by qRT-PCR. Infected-cell polypeptide 4 (ICP4) and ICP27 are two of the five immediate early transcriptional regulatory proteins that are expressed promptly upon HSV-1 infection [49]. ICP4 and ICP27 function to initiate viral DNA replication and to stimulate the transcription of early and late viral genes including *UL39*, which encodes the large subunit of ribonucleotide reductase [49]. The levels of all three gene transcripts were increased in *Tagap* ^−/−^ BMDMs compared to WT BMDMs (Figure 6C). Significantly more viral DNA was detected in *Tagap* ^−/−^ BMDMs as well (Figure 6D). Additionally, levels of viral protein products were compared between WT and Tagap^−/−^ BMDMs by immunobloting and results were consistent with the qRT-PCR data (Figure 6E). These findings suggest a role for Tagap in antiviral response.

To assess the influence of *Tagap* deficiency on innate immune signaling cascades, expression profiles of type I IFNs, pro-inflammatory cytokines and chemokines were measured in WT and *Tagap* ^−/−^ BMDMs upon HSV infection. As expected, *Tagap* ^−/−^ BMDMs were defective in several key mediators of the antiviral response, including IFN-β (Figure 6F). Consistent with reduced *Ifnb1* mRNA level, secreted IFN-β also decreased in *Tagap* ^−/−^ BMDMs after HSV infection (Figure 6G). These results suggest that Tagap plays a critical role in antiviral signaling pathways.

## Discussion

A number of studies have suggested a connection between genetic susceptibility to complex disease and viral infection [50]–[53]. However, there are only a handful of reports on specific virus-gene interactions and it remains unclear how many of the genes within T1D susceptibility loci are associated with antiviral immunity [54], [55]. Placing T1D genes into the viral defense pathway could help identify therapeutic entry points for T1D treatment as well as a more complete understanding of the environmental factors altering disease susceptibility. From the screen, we identified 14 candidate genes, the differential expression of which influences the antiviral activity of HSV-1-infected macrophage cells. However, whether these gene-virus interactions are sufficient to cause disease requires further investigation.

In an effort to identify novel host restriction factors against HSV-1 infection, many previous investigations focused on analysis of large-scale expression data and characterized the genes that are differentially regulated by IFNs and HSV-1 [56]–[58]. While this strategy was successful at identifying host restriction factors, a number of identified genes collaborate with IFN-induced genes to construct the antiviral network in host cells. Among the 14 candidates identified from the screen, only 5 were upregulated by IFN-γ and/or HSV-1. This result suggests that a number of antiviral host defense genes are constitutively expressed and their activity is only enhanced in the presence of antiviral cytokines such as IFN-γ. While our screening strategy takes an unbiased approach, due to the use of IFN-γ in the screen, the genes that function in late stage of viral suppression may be undervalued because the loss of these genes may be rescued or compensated by the activity of IFN-γ and its effector molecules. Regardless, our screen preserved the integrity of IFN pathways and thus examined the role of each candidate in the context of an intact antiviral response which creates a physiologically relevant system.

IFNs are the major cellular restriction factors fighting against viral infection. IFNs bind to their receptors and activate the downstream JAK-STAT signaling pathway that further leads to augmented antiviral responses. Among the 14 candidate genes identified from the genetic screen, *Tyk2*, *Ciita* and *IL-27*, are direct targets of IFN activation. Tyk2 belongs to the family of Janus kinases (JAKs) and is activated in response to type I IFNs [59]. Tyk2 phosphorylates the transcription factor Stat1 and leads to a functional type I IFN response [60]. Ciita, class II transactivator, is a well-studied positive regulator for expression of class II MHC genes upon stimulation, and consistent with our results, studies have shown that IFN-γ induces expression of Ciita in a Stat1-dependent manner [61]. Given the functional significance of MHC class II molecules in initiation and maximization of the adaptive immune response, Ciita acts as a bridge between innate and adaptive immunity, thus playing a critical role in controlling viral infection. Similar to type I IFNs, IL-27 is also capable of activating the JAK-STAT pathway and inducing expression of inflammatory cytokine genes in macrophages [62], [63]. These findings suggest that T1D genes within risk loci function in a common signaling pathway, which is composed of IFNs/IL-27, JAK/Tyk2, Stat1, Ciita, and the downstream effectors. Besides the connection with the JAK-STAT signaling pathway, a subgroup of the candidate genes (*Tyk2*, *Cdk2*, *Prkd2*, *Sult1a1*, *Rbm17*, and *Ciita*) are enriched based on DAVID analysis for nucleotide binding, including adenyl nucleotide/ribonucleotide binding and ATP binding, a pathway important for antiviral signaling (Table S3). Although many of the candidate genes identified from the genetic screen do not seem to possess direct antiviral properties, 12 of them, with the exception of *Il27* and *Dtx3*, have been shown to be differentially expressed in peripheral blood mononuclear cells isolated from Ebola virus-infected nonhuman primates [64]. This result suggests that these genes may play a role in antiviral pathways or virally induced cellular processes. Further investigation is required to delineate the function of individual candidate genes in the context of viral infection.

Previous reports have shown that IL-27 is induced by a variety of viruses, including HIV, HBV, influenza A virus, murine gammaherpesvirus 68 (HV-68), and Theiler's virus [65]–[68]. In HIV infection, IL-27 mediates viral suppression in human macrophages in a mechanism similar to IFN-α, which induces the expression of downstream antiviral molecules such as the family of APOBEC cytidine deaminases [42]. In our experimental system, IL-27 alone is not sufficient to induce antiviral immunity, suggesting that host restriction factors induced by IFN-γ cooperate with IL-27 to boost antiviral immunity to HSV-1 infection. Recently, IFN-λ1, a type III IFN, was shown to be co-induced with IL-27 by HBV infection and to cooperate with IL-27 to limit HBV replication in HepG2 cells [69]. Unfortunately, the IFN-λ1 gene is a pseudogene in mice [70] and examination of the other two members of the type III IFN family, IFN-λ2 and -λ3, suggested that the coordinated regulation of IL-27 and IFN-λ does not seem to operate in murine macrophage cells upon HSV-1 infection. Taken together, these data suggest that IL-27 can work in concert with a variety of IFNs to mediate host defense against a plethora of viruses.

Single nucleotide polymorphisms (SNPs) within the *Tagap* locus have been identified as a shared risk factor for Crohn's disease and celiac disease, while other SNPs within this locus have been associated with protection from rheumatoid arthritis and T1D [71], [72]. Tagap is highly expressed in immune cells, including B cells, T cells, dendritic cells, natural killer cells, and monocytes; however, little is known about the function of Tagap in host defense. Our data suggest that Tagap plays a role in regulating key antiviral cytokines. However, future studies will be important to define the mechanism of action of Tagap given its broad association with complex diseases.

In summary, our unbiased loss-of-function genetic screen identified genes within T1D susceptibility loci that work in concert with IFN signaling pathways to protect host cells from detrimental viral infection, thus preventing improper immune responses leading to inflammation and autoimmunity. Better understanding of the mechanisms of the gene-plus-virus interaction will provide new approaches to design therapies to treat complex diseases.

## Materials and Methods

### Ethics statement

Mice used in this study were maintained in specific-pathogen-free facilities at Massachusetts General Hospital (Boston, MA). All animal studies were conducted under protocols approved by the Institutional Animal Care and Use Committee (IACUC) at Massachusetts General Hospital. These mice were bred and experiments were approved under protocol 2007N000045.

### Cells, viruses, mice, and reagents

RAW 264.7 cells (TIB-71) and Vero cells (CCL-81) were obtained from ATCC and cultured in DMEM (Gibco) supplemented with 10% fetal bovine serum (FBS) (Hyclone), 1% GlutaMAX (Life Technologies) and 20 µg/ml gentamycin sulfate (Sigma-Aldrich). IRF and NF-κB/IRF reporter cells were generated by transducing RAW 264.7 cells with lentiviral-based firefly luciferase expression constructs under the control of either IRF consensus sequence (GAAA ACGAAACT ) and/or NF-κB consensus sequence (GGGA AATTCC ). Recombinant IL-27 was purchased from eBioscience and used at a concentration of 50 ng/ml.

The *Tagap* ^−/−^ mouse was a kind gift from Dr. Bernhard G. Herrmann [73]. As reported in the original publication by Bauer et al., the accession number for the gene targeted in this knockout model is NM_145968, which corresponds to the *Tagap* gene. However, Bauer et al. refer to this gene as *Tagap1*. To clarify the gene targeted in these mice, we developed a quantitative RT-PCR method and showed that the targeted mice lacked *Tagap* mRNA, consistent with the accession number referenced in the original publication (Figure S5). See also MGI ID 3615484 for gene information and MGI ID 3603008 for mouse strain information.

BMDMs were generated by culturing mouse bone marrow cells in RPMI (Gibco) supplemented with 10% FBS, 1% GlutaMAX, 20 µg/ml gentamycin sulfate and 20 ng/ml M-CSF (Peprotech) for 6 days.

HSV-GFP were propagated and titrated in Vero cells. Briefly, Vero cells were infected with HSV-GFP at low multiplicity of infection (MOI). Culture supernatant was collected when >95% cytopathic effect observed and cell debris was removed by centrifugation. The viral stock was aliquoted and stored at −80°C until use. Virus titer was determined by plaque assay and crystal violet stain. The virus stock contained 3×10^6^ plaque-forming unites (PFU)/ml. In experiments, cells were infected with HSV-GFP at a MOI of 0.5 or as specified in figure legends.

Sendai virus was purchased from ATCC (VR-105). In experiments, cells were infected with Sendai virus at a MOI of 1.

### Genetic screen

161 genes within T1D susceptibility loci were selected from genes in T1Dbase (http://www.t1dbase.org). We filtered genes based on (1) those genes closest to T1D SNPs and (2) expression of these genes in immune cells based on data from publicly available databases. The lentivirus-based shRNA library containing 827 shRNA sequences against these 161 genes was obtained from the RNAi Consortium (Broad Institute). RAW 264.7 cells were seeded in 96-well flat-bottom plate on day 0 and transduced with lentiviruses on day 1. On day 2, puromycin (Sigma-Aldrich) was added to cell culture media at a final concentration of 3 µg/ml. Cells were fed once with puromycin-containing media on day 5. On day 6, cells were split and approximately 0.5×10^5^ cells per well were seeded in 96-well glass bottom plates (In Vitro Scientific) and stimulated with mouse IFN-Γ (Shenandoah Biotechnology) at a final concentration of 10 ng/ml. On day 7, cells were infected with HSV-GFP at a MOI of 0.5 for 16 h before fixing with 4% paraformaldehyde (PFA) (Santa Cruz Biotechnology). Nuclei were stained with Hoechst 33342 nucleic acid stain (Life Technologies) for 10 min. Images were collected with 2 emission wavelengths, GFP for virus and Hoechst for host cell nuclei, by automated fluorescent microscopy (Molecular Devices). Images for six microscopic fields per well were captured and analyzed using a custom pipeline generated from CellProfiler cell image analysis software [74]. The total number of cells per field was determined by the number of Hoechst-labeled nuclei. HSV-GFP infection efficiency per field was determined by the percentage of GFP-positive cells. The HSV-GFP infection efficiency for each individual sample that was transduced with different lentiviral shRNA was determined by the average percentage of GFP-positive cells from the six images collected from the same well. Data obtained from each 96-well plate were range-normalized by scaling between 0 (0%) (computed by the median of wells transduced with irrelevant shRNA controls) and 1 (100%) (computed by median of wells transduced with positive control shRNA). Range normalization was done on per-plate basis, then all normalized data (expressed as percentage within the range) were pooled and Z scores calculated individually from the distribution of normalized irrelevant shRNA controls. shRNAs with a Z score equal or greater than 3 was considered a “hit” of the screen.

### RNA and DNA isolation and quantitative real-time PCR

RNA isolation was performed using a NucleoSpin 96 RNA Isolation Kit (Macherey-Nagel) or RNeasy Mini Kit (Qiagen) according to the manufacturers' instructions. cDNA was synthesized using the iScript cDNA synthesis kit (Bio-Rad) and qRT-PCR was performed using iQSYBRGreen super mix (Bio-Rad) with 300 nM forward and reverse primers in a CFX386 Real-Time PCR System (Bio-Rad). Relative levels of target mRNA were normalized to Gapdh mRNA. Viral genomic DNA was isolated using QuickExtract DNA Extraction Solution (Epicentre). ICP27 DNA content was measured by qRT-PCR and normalized to ribosomal protein large P0 (Rplp0). Primers used are listed in Table S4.

### ELISA

Cell culture supernatants were collected 12 h after stimulation. The concentration of IFN-β was measured using VeriKine Mouse Interferon Beta ELISA kit (PBL Assay Science) according to the manufacturer's instructions.

### Flow cytometry

HSV-infected BMDMs were harvested at 12 h as indicated in figure legends. Following infection, plates were spun at 500×g and supernatants collected for ELISA. Cells were washed once with PBS and stained with Live/Dead fixable stain (Invitrogen, Life Technologies) according to the manufacturer's instructions. Cells were harvested by scraping, blocked with Fc block, and stained with mouse anti CD11b (eBioscience). Cells were then fixed using Foxp3/Transcription Factor intracellular staining kit (eBioscience) according to the manufacturer's instructions. GFP was detected using Alexa Fluor 647-conjugated anti-GFP (Life Technologies). Samples were acquired using an LSR OO flow cytometer (BD Biosciences) and post-acquisition analysis was performed using FlowJo software (Tree Star Inc.)

### Whole cell extract and Western blotting

Cells were lysed in RIPA buffer (25 mM Tris⋅HCl pH 7.6, 150 mM NaCl, 1% NP-40, 1% sodium deoxycholate, 0.1% SDS) supplemented with Protease Inhibitor Cocktail (Roche) and incubated on ice. The mixture was vortex mixed every 3 min for 15 min and centrifuged at 13,000 rpm for 10 min at 4°C. The supernatant was harvested and protein concentration was measured by BCA Protein Assay Kit (Thermo Scientific Pierce). After separating on sodium dodecyl sulfate (SDS)–polyacrylamide gel, proteins were visualized using antibodies specific to HSV-1 ICP4 (H943) and ICP27 (vP-20) (Santa Cruz Biotechnology). β-actin levels were visualized as a control (Sigma-Aldrich). A detailed procedure for Western blotting has been described previously [75].

### Luciferase reporter assay

Cells were stimulated as described in the text. On the day of experiment, PrestoBlue Cell Viability Reagent (Life Technologies) was added to cells and incubated for 20 min. Absorbance was measured in a SpectraMax M5 Microplate Reader (Molecular Devices). After reading the plate, culture supernatants were removed and luciferase activity was determined using SteadyLite plus Reporter Gene Assay System (PerkinElmer) in TopCount NXT Microplate Luminescence Counter (PerkinElmer). Relative reporter activity was normalized to cell viability determined earlier by PrestoBlue viability assay.

### Data analysis

All experiments were repeated independently as indicated in the figure legends. Averaged or representative results from all repeats are shown. Statistical analyses are described in figure legends; significance was defined as *P*<0.05.

Gene ontology enrichment analysis was performed using the Database for Annotation, Visualization and Integrated Discovery (DAVID) [76], [77].

## Supporting Information

Figure S1 **Cell Profiler image analysis pipeline identifies GFP-positive nuclei as HSV-1-infected RAW 264.7 cells.** RAW 264.7 cells were seeded in 96-well glass bottom plates at 0.5×10^5^ cells/well and infected with HSV-GFP at an MOI of 1. After 16 h, cells were fixed and stained with Hoechst 33342 nucleic acid stain. Shown are representative images collected by fluorescent microscopy. A CellProfiler analysis pipeline was applied to calculate the efficiency of viral infection. Hoechst stain for dsDNA was used to identify cells and GFP expression indicates viral infection. The number of GFP-positive nuclei (cells) was determined for each image.
(PDF)

Figure S2 **Knockdown of antiviral genes increases HSV-GFP infectivity.** (**A**) RAW 264.7 cells were seeded in 96-well flat bottom plates at low density (0.1×10^5^ cells/well) and transduced with lentivirally encoded shRNAs against three key players involved in antiviral pathways (Ticam2, Ifngr1, and Ifngr2) or non-targeting controls (lacZ and luciferase). After four days of puromycin selection, cells were split, seeded in 96-well glass bottom plates at approximately 0.3×10^5^ cells/well and stimulated with IFN-γ overnight before infecting with HSV-GFP at an MOI of 0.5. The average percentage of GFP-positive cells was calculated from six individual images per sample. Data shown are representative of 3 independent experiments. ***P<0.0001. Ticam, Ifngr1, and Ifngr2 were each compared to individual negative controls by t-tests; Fisher's exact method was used to combine individual *P* values and generate an overall *P* value for each tested gene. **(B)** Representative images of cells transduced with irrelevant control shRNA against lacZ or positive control shRNA against Ifngr1. The total number of cells per image was determined by Hoechst nuclei acid stain and number of HSV-GFP infected cells was determined by GFP expression. Efficiency of viral infection was then calculated as the percentage of GFP-positive cells. Knockdown of Ifngr1 in RAW 264.7 cells results in approximately 3.5 fold increase in viral infectivity.
(PDF)

Figure S3 **Relative expression of candidate genes in RAW 264.7 cells after transduction with lentivirally encoded shRNA.** Cells were seeded and transduced with different targeting shRNAs as described in Figure S2. After 96 h of selection in puromycin-containing media, cells were stimulated with IFN-γ (10 ng/ml) for 16 h. After stimulation, cells were harvested and RNA was isolated. qRT-PCR was performed to measure the knockdown of individual candidate gene. Expression of each candidate gene in cells transduced with irrelevant shRNA is set to 1. Expression of the same gene in cells transduced with targeting shRNA is calculated as relative to the irrelevant control. *Gapdh* was used as an internal control for normalization. Results were derived from two independent experiments.
(PDF)

Figure S4 **IL-27 enhances the activity of NF-κB and IRF3/7 in Sendai virus-infected RAW 264.7 cells.** NF-κB/IRF and IRF reporter cells were seeded in 96-well clear bottom plates at 1×10^5^ cells per well and either left uninfected or infected with Sendai virus (SeV) at an MOI of 0.5 in the presence or absence of recombinant IL-27 (50 ng/ml). After 16 h, cells were lysed and luciferase activities were measured. The reporter luciferase activity in untreated cells is normalized to 1. Data shown are results from 3 independent experiments; error bars represent standard deviation. ***P<0.0001 by unpaired two-tailed t-tests.
(PDF)

Figure S5 ***Tagap*** **and** ***Tagap1*** **expression in** ***Tagap*** **KO mice.** **(A)** Primers used for verifying expression of *Tagap* and *Tagap1* in KO mice. **(B–C)** Expression of *Tagap1* **(B)** and *Tagap* **(C)** mRNA in total splenocytes from WT, heterozygous (Het), and KO mice. In (C), expression levels of the WT allele are normalized to *Tagap* expression in WT mice; expression levels of the KO allele are normalized to *Tagap* expression in KO mice. The targeted (KO) mice express *Tagap1* at levels similar to WT mice, whereas WT, Het, and KO mice display patterns of expression consistent with *Tagap* gene targeting. Thus, the mice described here lack *Tagap* expression.
(PDF)

Table S1 **List of targeting shRNAs used in the primary genetic screen.** This table includes target sequences, target gene ID, target gene symbol, region of the gene (CDS, coding sequence; UTR, untranslated region), and estimated viral titer of lentiviral particles for each shRNA hairpin.
(XLSX)

Table S2 **Percentile rank and Z score of individual shRNA hairpins that scored in the primary genetic screen.**
(XLSX)

Table S3 **Gene ontology enrichment analysis of confirmed hits from secondary screens.**
(XLSX)

Table S4 **List of PCR primer sequences used in this study.**
(XLSX)
