# Supplementary figures and images for: An Image-Based Genetic Assay Identifies Genes in T1D Susceptibility Loci Controlling Cellular Antiviral Immunity in Mouse
Source: PLoS One. 2014 Sep 30;9(9):e108777. doi: 10.1371/journal.pone.0108777 (PMC4182575; doi:10.1371/journal.pone.0108777)

# Figure S1

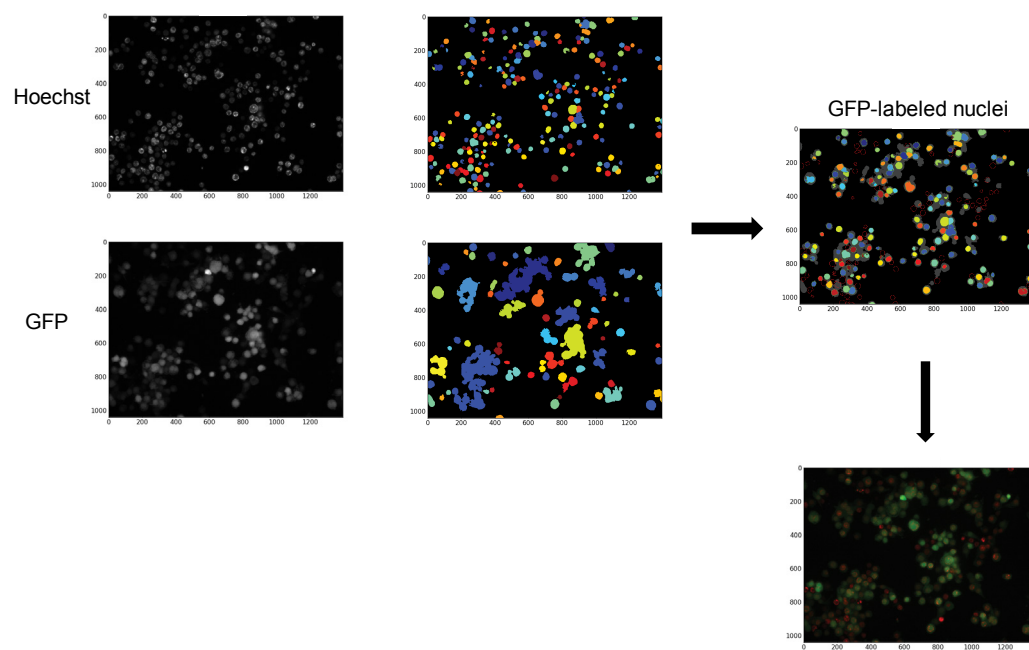

Supplement: Figure S1 — Cell Profiler image analysis pipeline identifies GFP-positive nuclei as HSV-1-infected RAW 264.7 cells. RAW 264.7 cells were seeded in 96-well glass bottom plates at 0.5×10^5^ cells/well and infected with HSV-GFP at an MOI of 1. After 16 h, cells were fixed and stained with Hoechst 33342 nucleic acid stain. Shown are representative images collected by fluorescent microscopy. A CellProfiler analysis pipeline was applied to calculate the efficiency of viral infection. Hoechst stain for dsDNA was used to identify cells and GFP expression indicates viral infection. The number of GFP-positive nuclei (cells) was determined for each image. (PDF) [file pone.0108777.s001.pdf]

# Figure S2

**A**

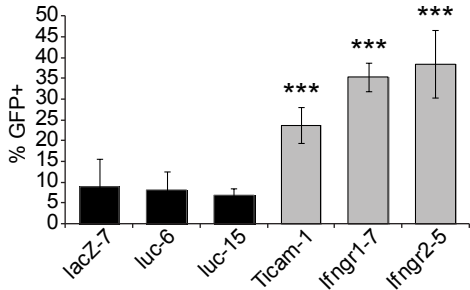

**B**

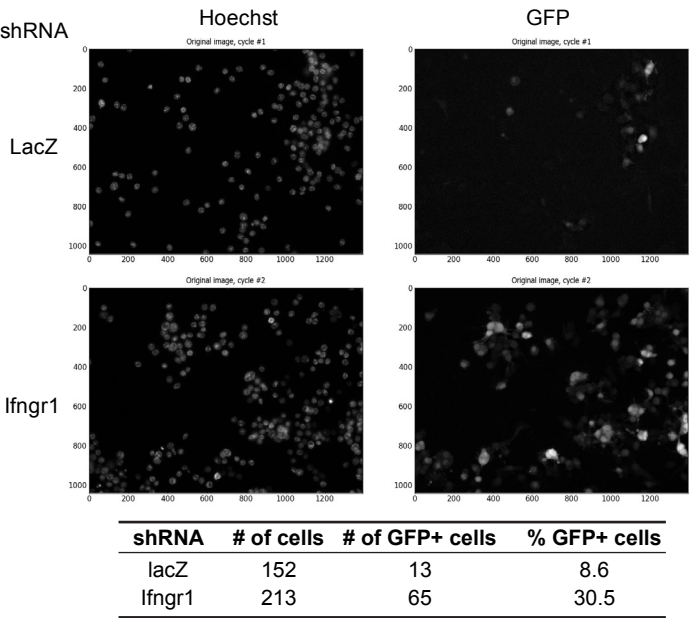

Supplement: Figure S2 — Knockdown of antiviral genes increases HSV-GFP infectivity. (A) RAW 264.7 cells were seeded in 96-well flat bottom plates at low density (0.1×10^5^ cells/well) and transduced with lentivirally encoded shRNAs against three key players involved in antiviral pathways (Ticam2, Ifngr1, and Ifngr2) or non-targeting controls (lacZ and luciferase). After four days of puromycin selection, cells were split, seeded in 96-well glass bottom plates at approximately 0.3×10^5^ cells/well and stimulated with IFN-γ overnight before infecting with HSV-GFP at an MOI of 0.5. The average percentage of GFP-positive cells was calculated from six individual images per sample. Data shown are representative of 3 independent experiments. ***P<0.0001. Ticam, Ifngr1, and Ifngr2 were each compared to individual negative controls by t-tests; Fisher's exact method was used to combine individual P values and generate an overall P value for each tested gene. (B) Representative images of cells transduced with irrelevant control shRNA against lacZ or positive control shRNA against Ifngr1. The total number of cells per image was determined by Hoechst nuclei acid stain and number of HSV-GFP infected cells was determined by GFP expression. Efficiency of viral infection was then calculated as the percentage of GFP-positive cells. Knockdown of Ifngr1 in RAW 264.7 cells results in approximately 3.5 fold increase in viral infectivity. (PDF) [file pone.0108777.s002.pdf]

Figure S3

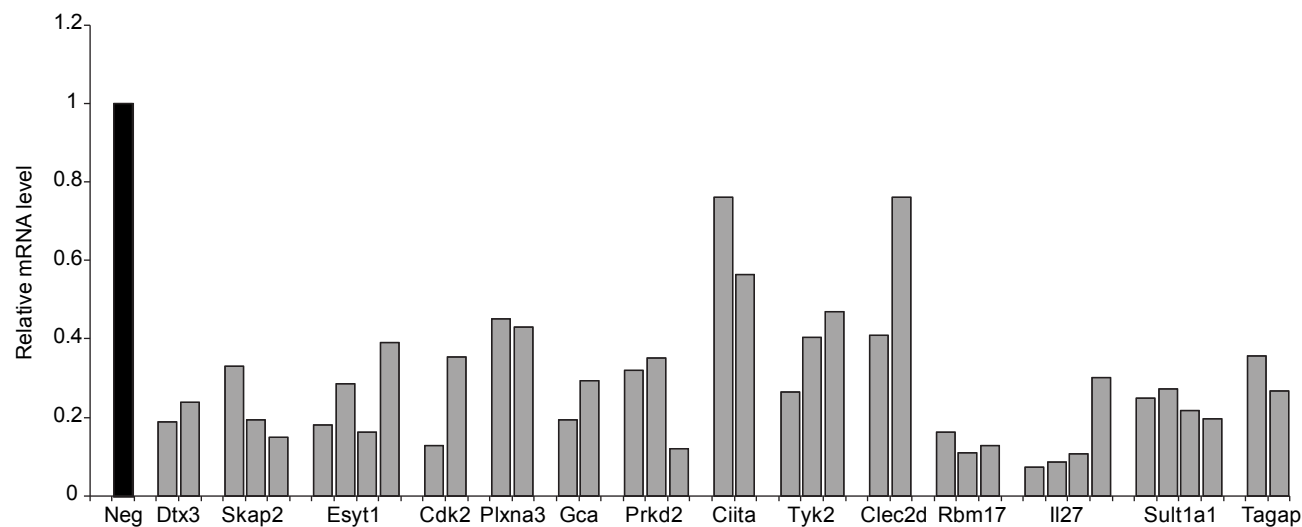

Supplement: Figure S3 — Relative expression of candidate genes in RAW 264.7 cells after transduction with lentivirally encoded shRNA. Cells were seeded and transduced with different targeting shRNAs as described in Figure S2. After 96 h of selection in puromycin-containing media, cells were stimulated with IFN-γ (10 ng/ml) for 16 h. After stimulation, cells were harvested and RNA was isolated. qRT-PCR was performed to measure the knockdown of individual candidate gene. Expression of each candidate gene in cells transduced with irrelevant shRNA is set to 1. Expression of the same gene in cells transduced with targeting shRNA is calculated as relative to the irrelevant control. Gapdh was used as an internal control for normalization. Results were derived from two independent experiments. (PDF) [file pone.0108777.s003.pdf]

Figure S4

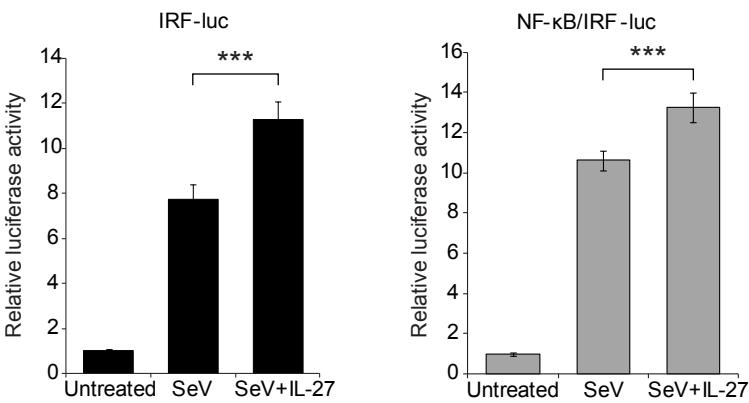

Supplement: Figure S4 — IL-27 enhances the activity of NF-κB and IRF3/7 in Sendai virus-infected RAW 264.7 cells. NF-κB/IRF and IRF reporter cells were seeded in 96-well clear bottom plates at 1×10^5^ cells per well and either left uninfected or infected with Sendai virus (SeV) at an MOI of 0.5 in the presence or absence of recombinant IL-27 (50 ng/ml). After 16 h, cells were lysed and luciferase activities were measured. The reporter luciferase activity in untreated cells is normalized to 1. Data shown are results from 3 independent experiments; error bars represent standard deviation. ***P<0.0001 by unpaired two-tailed t-tests. (PDF) [file pone.0108777.s004.pdf]

# Figure S5

A

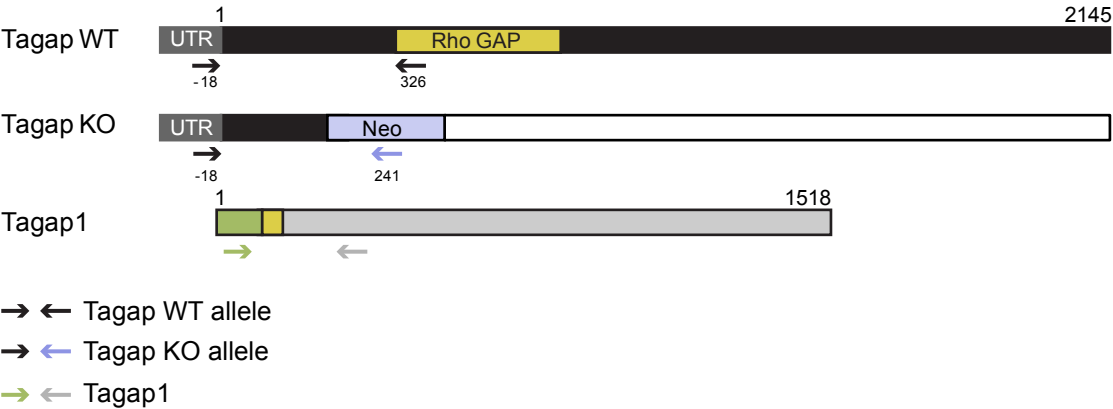

B

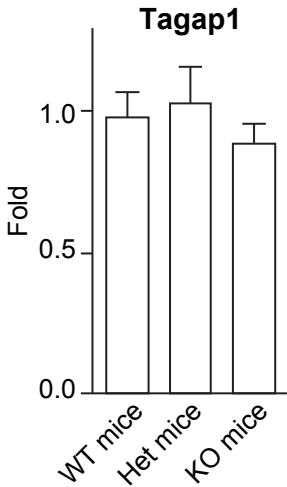

C

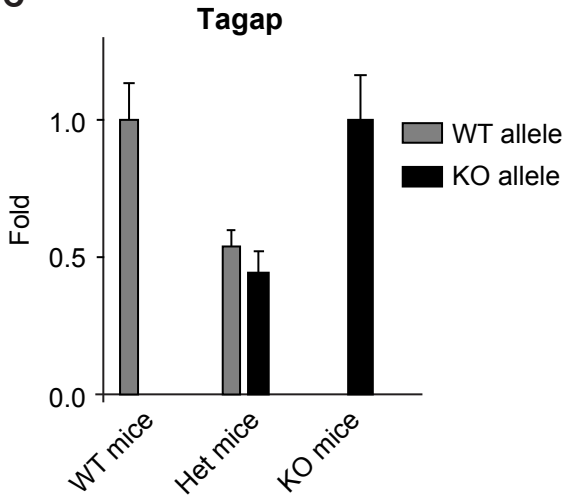

Supplement: Figure S5 — Tagap and Tagap1 expression in Tagap KO mice. (A) Primers used for verifying expression of Tagap and Tagap1 in KO mice. (B–C) Expression of Tagap1 (B) and Tagap (C) mRNA in total splenocytes from WT, heterozygous (Het), and KO mice. In (C), expression levels of the WT allele are normalized to Tagap expression in WT mice; expression levels of the KO allele are normalized to Tagap expression in KO mice. The targeted (KO) mice express Tagap1 at levels similar to WT mice, whereas WT, Het, and KO mice display patterns of expression consistent with Tagap gene targeting. Thus, the mice described here lack Tagap expression. (PDF) [file pone.0108777.s005.pdf]
